# A large database linking the rumen bacterial composition and milk traits in Lacaune sheep

**DOI:** 10.1038/s41597-022-01912-3

**Published:** 2023-01-07

**Authors:** Guillermo Martinez Boggio, Christel Marie-Etancelin, Jean-Marie Menras, Regis Tomas, Marie-Luce Chemit, Béatrice Gabinaud, Géraldine Pascal, Annabelle Meynadier

**Affiliations:** 1 GenPhySE, Université de Toulouse, INRAE, ENVT, F-31326 Castanet Tolosan, France; 2 INRAE, Experimental Unit of La Fage, F-12250 Saint-Jean et Saint-Paul, France

**Keywords:** Metagenomics, Animal physiology

## Abstract

Ruminants are able to produce food for human consumption from plants, thanks to rumen bacteria. Bacteria are able to transform feed to microbial proteins and to biohydrogenate unsaturated fatty acids, contributing directly to fine milk composition. The database consists of daily records of milk yield, somatic cell score and 17 milk components such as fatty acids and proteins from 795 Lacaune dairy ewes. Ruminal samples were extracted from ewes using a gastric tube and sequenced to determine the bacterial composition by metabarcoding 16S rRNA gene on a next-generation sequencing platform. From bioinformatics analysis, 9,536,442 sequences were retained and re-grouped into 2,059 affiliated OTUs, represented by 751 to 168,617 sequences. Overall, 2,059 OTUs from 795 samples were attributed to 11 phyla. The most representative phyla were *Bacteroidota* (50.6%) and *Firmicutes* (43.6%), and the most abundant families were *Prevotellaceae* (37.9%), *Lachnospiraceae* (18.1%), *Ruminococcaceae* (8.97%). Both shared datasets will be useful for researchers to study the link between rumen bacteria and milk traits and to propose solutions to improve animal production and health.

## Abstract (summary)

**Table:** 

Measurement(s)	milk yield • milk somatic cell quantity • mid-infrared spectroscopy • Fat content • Protein content • milk fatty acid C4:0 amount • milk fatty acid C6:0 amount • milk fatty acid C8:0 amount • milk fatty acid C10:0 amount • milk fatty acid C12:0 amount • milk fatty acid C16:0 amount • milk fatty acid cis-9-C18:1 amount • milk fatty acid cis-9,trans-11-C18:2 amount • milk fatty acid C18:3(n-3) amount • milk alpha-S2-casein amount • milk alpha-S1-casein amount • milk beta-casein amount • milk kappa-casein amount • milk alpha-lactalbumin amount • milk beta-lactoglobulin amount
Technology Type(s)	Weighted milk quantity per day • Fossomatic cell counter • Milko-ScanTM FT6000 • Prediction from mid-infrared spectroscopy
Factor Type(s)	Genetic lines • Year of sampling • Batch • Parity • Days in milk • Litter size • Total number of sequences • Sampling order • Sampling hour
Sample Characteristic - Organism	Ovis aries
Sample Characteristic - Environment	Experimental Unit - Indoor conditions
Sample Characteristic - Location	La Fage, 12250 Sant-Jean-et-Saint-Paul

## Background & Summary

Ruminants have the particular capacity to transform plant fiber into usable energy for the production of human food. This is achieved through ruminal symbiosis with a diverse microbial community, mainly composed of bacteria that transform dietary carbohydrates, proteins and complex lipids into volatile fatty acids, lactate, microbial proteins and vitamins^1^. To study rumen bacterial communities, one of the most commonly used techniques is targeting sequencing of the 16S rRNA gene of prokaryotes. 16S rRNA sequencing is a powerful and robust way to study large cohorts of animals^2^, which combined with zootechnical traits allows the study of associations between rumen microbiota and the livestock production.

A large dataset was generated through an experiment involving 795 mid-lactation adult dairy ewes from which we collected rumen and milk samples for five years under the same housing conditions. Rumen samples were obtained directly from the rumen with a gastric tube and then sequenced by the aforementioned targeting sequencing of the 16S rRNA gene using a next-generation sequencing platform. The bioinformatics process of the resulting millions of DNA sequences generated 2,059 operational taxonomic units (OTUs) as final data, whose taxonomic affiliation was retrieved from the SILVA database^3^. Milk samples were obtained from each ewe along with the rumen sample. From daily milk samples, the milk yield and somatic cell counts were recorded, and mid-infrared (MIR) spectra were retrieved to predict fat and protein contents and fatty acid and protein profiles.

The dataset presented was partially analyzed by Martinez Boggio *et al*.^4^, but here we describe in detail the experimental conditions.

## Methods

Data were obtained from the INRAE Experimental Unit of La Fage (UE 321 agreement A312031, Roquefort, France) between 2015 and 2019. The dataset consisted of 795 adult Lacaune dairy ewes in mid-lactation (109 ± 16.0 days in milk) under the same housing conditions. The ewes (weighing 76.9 ± 8.68 kg) had two or more lactations and lambed one or more lambs.

### Animal management

The protocol used for animal experimentation received approval from the Ministère de l’Enseignement Supérieur de la Recherche et de l’Innovation – Animal ethics committee with the following approval number APAFIS#6292–2016080214271984 v8. The genetic structure of the INRAE La Fage flock includes four independent divergent genetic lines with two selected for milk somatic cell score (SCS) and two selected for milk persistency (PERS).

Genetic selection of SCS lines was initiated in 2003^5^ based on the estimated breeding values (EBV) for milk SCS (log-transformed somatic cell count) of sires of the whole Lacaune population. Each year, two groups of dams with extreme EBVs for SCS were mated with extreme males, and the female offspring were segregated into a high-SCS line (SCS+) and a low-SCS line (SCS-). This selection was demonstrated by Rupp *et al*.^6^ to produce ewes with susceptibility/resistance to natural clinical and subclinical mastitis. Genetic selection of PERS lines was based on EBV of Lacaune sires belonging to the whole Lacaune population based on the coefficient of variation in milk production on the testing day. The selection started in 2009, with extreme sires of the whole population mated to extreme La Fage dams for milk persistency. Each year, two extreme groups of ewes were created, one with high persistence (PERS+) and one with low persistence (PERS-), for milk production.

Furthermore, ewes selected for the gene suppressor of cytokine signaling 2 (Socs2)^7^ were also included in the dataset. That population was derived from the oldest SCS lines and is currently selected for Socs2 to increase the mutant allele frequency in the experimental population to investigate possible associations with traits other than SCS. The dataset contains information from 795 ewes, including 94 from SCS+, 204 from SCS-, 200 from PERS+ and 202 from PERS-, and 95 under the Socs2 selection.

All ewes were raised indoors, and to meet their requirements, they were fed the same mixed ration of on average 90.0% meadow hay and silage plus 10.0% barley (on a gross matter basis) supplemented with approximately 150 g of a commercial protein concentrate (38.0% of crude protein on dry matter basis) distributed in the milking parlor. Ration distribution was collective and takes into account a 15.0% refusal level to ensure that each individual was fed *ad libitum*. Because of variations in forage, the adjustment of the percentage of concentrates and forages was done each year according to the feeding value of the forages to cover the needs of the ewes, which therefore received the same amounts of nutrients over the five years. On average, over the five years, the ewes ingested 3.27 kg of dry matter in total, comprised of 16.0% crude protein and 30.0% crude fiber. The authors advise using their data corrected for the year effect as in Martinez Boggio *et al*.^4^.

### Rumen sampling

The rumen sampling was performed from each ewe using a gastric tube consists of a flexible silicon PVC tube (5 mm of wall thickness and 8 mm of internal diameter; Stomaflex, Genia, France) with a rounded open tip with a lateral eye to prevent solid material from accumulating in the tube, and was rinsed with clean water and drained between samplings to avoid cross-contamination. The animal immobilization was performed with a special cage adapted for ewes, and sampling was performed by 2 competent sheep keepers: one holding the animal’s head in a slightly raised position and the second inserting the gastric tube into the animal’s throat through a bit to prevent the tube from being bitten. The vacuum pump is only activated when the tube is fully descended into the rumen and is then switched off before the tube is raised to avoid collecting saliva. On average, we collected 30 ml of rumen samples per animal with a liquid fraction and small solid particles. All samples were first subjected to a visual examination to ensure that they were not contaminated by saliva or blood. Furthermore, to avoid dilution of samples by feed or water, the animals did not have access to feed for ten hours and to water for two hours prior to sampling. Finally, we directly aliquoted the rumen samples and froze them in liquid nitrogen and stored them at −80 °C. We performed the rumen sampling within three days of milk sampling, so that rumen composition and milk composition are associated in time and space.

### Targeting 16S rRNA gene sequencing

Total DNA from 80.0 μL of ruminal sample was extracted and purified using the QIAamp DNA Stool Mini Kit (Qiagen Ltd, West Sussex, UK) according to the manufacturer’s instructions, with a previous bead-beating step in a FastPrep instrument (MP Biomedicals, Illkirch, France).

The 16S rRNA V3-V4 regions of the samples were amplified (first PCR: 30 cycles) from purified genomic DNA with the primers forward F343 (50–CTTTCCCTACACGACGCT. CTTCCGATCTACGGRAGGCAGCAG–30^8^) and reverse R784 (50–GGAGTTCAGACG. TGTGCTCTTCCGATCTTACCAGGGTATCTAATCCT–30^9^). As Illumina MiSeq technology enables 250 bp paired-end reads, we obtained overlapped reads that generated extremely high-quality (less than 2% error rate), full-length reads of the entire V3 and V4 regions in a single run. Single multiplexing was performed using a 6 bp index, which was added to R784 primer, during a second round of PCR with 12 cycles with home-made primers including also Illumina adapters: forward (AATGATACGGCGACCACCGAGATCTACACTCTTTCCCTACACGAC) and reverse (CAAGCAGAAGACGGCATACGAGATGTGACTGGAGTTCAGACGTGT). The resulting PCR products were purified and loaded onto a next-generation sequencing platform (MiSeq System, Illumina, San Diego, CA, USA) at the Genomic and Transcriptomic Platform (INRAE, Toulouse, France) according to the manufacturer’s instructions. This process was repeated each year between 2015 and 2019, but in the first three years, the sequencing process was carried out at different times, so the samples were not sequenced in the same batch.

### Bioinformatics process

The Genomic and Transcriptomic Platform (INRAE, Toulouse, France) delivered the results in the form of ready-to-use sequences, *i.e*., demultiplexed and merged sequences. Demultiplexing allowed us to assign each paired-end read to its sample based on the previously integrated index. The initial number of DNA sequences was 20,853,771, with a range of 3,944 to 60,761 DNA sequences per sample.

**Fig. 1:**
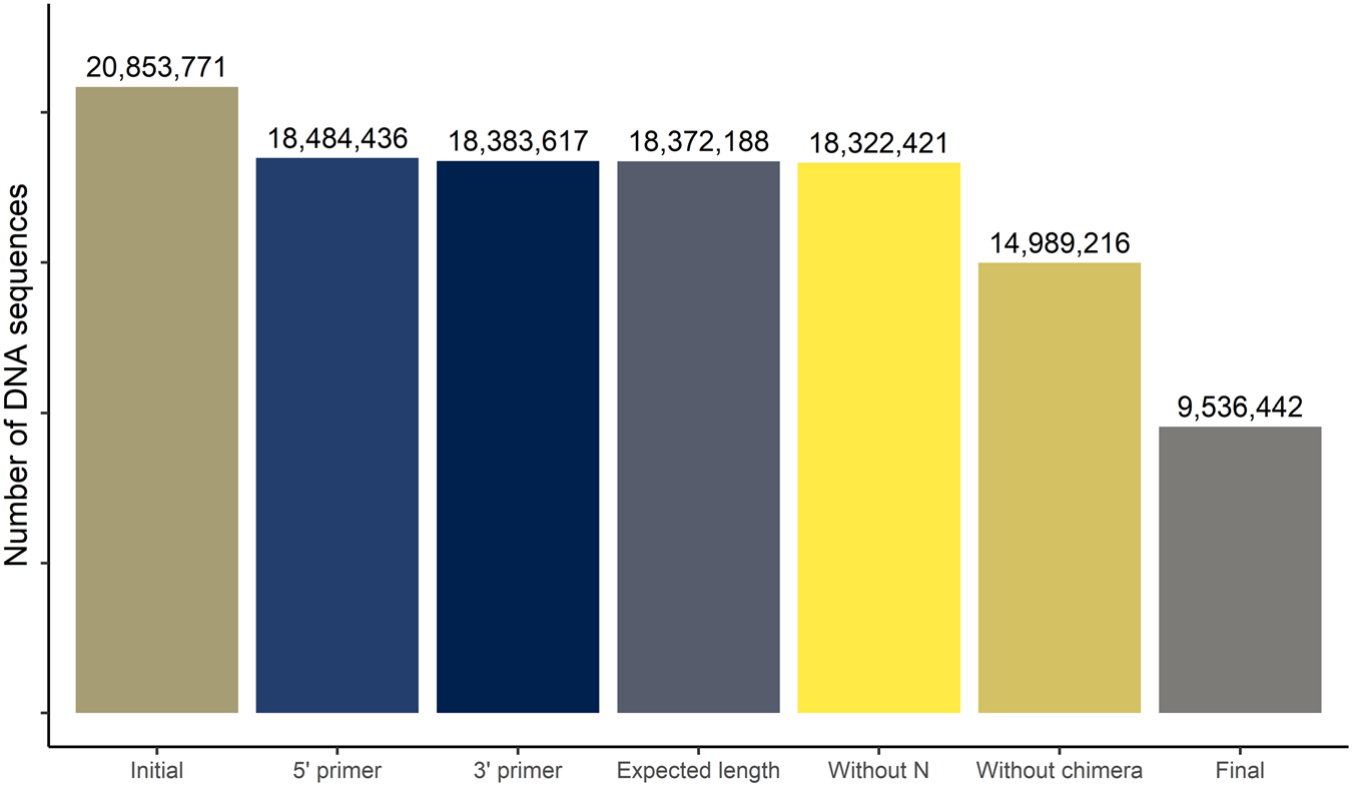
Number of DNA sequences during the sequencing data process. The 5′ primer and 3′ primer correspond to the number of sequences with these primers, expected length are the sequences with a length between 300 and 550 nucleotide pairs, without N corresponds to sequences with no aberrant nucleotides, without chimera corresponds to sequences without chimeric sequences, and Final is the final number of sequences after to apply the filters.

The bioinformatics process of DNA sequences was performed using the FROGS 3.0 pipeline^10^ according to the following procedure. The first step consists of read preprocessing, which will allow a first rough cleaning of the sequences by removing sequences presenting a primer mismatch, displaying an unexpected length, *i.e*., shorter than 300 bp or longer than 500 bp, that contain at least one ambiguous base. Approximately 12.0% of the sequences were discarded by this first filter (Fig. 1). Then, sequences were re-grouped by clustering with Swarm^11^ inside FROGS. We chose the parameters for a distance equal to 1 and the fastidious option (*--distance 1--fastidious*) as recommended in Swarm v2. During sequencing of relatively close amplicons, it was common for chimeras to form during PCR cycles. The next step in our process therefore allowed us to eliminate them. This indispensable step caused a loss of 18% of sequences compared to the raw data (Fig. 1) and 41.0% of OTUs (2,604,793 OTUs) compared to the initial OTUs of 6,394,941. Nevertheless, there were still artifacts from PCR and sequencing, for example, singletons. Therefore, in the following step, we chose to apply a strong filter by removing OTUs with abundances lower than 0.005% of total sequences^12^. As a result of the bioinformatics process, we retained 9,536,442 DNA sequences, representing 46.0% of the initial total DNA sequences (Fig. 1), and 2,059 OTUs, representing 0.03% of initial OTUs. The final step in the process was to give each OTU a taxonomic affiliation using the SILVA database (version 138)^3^.

**Fig. 2:**
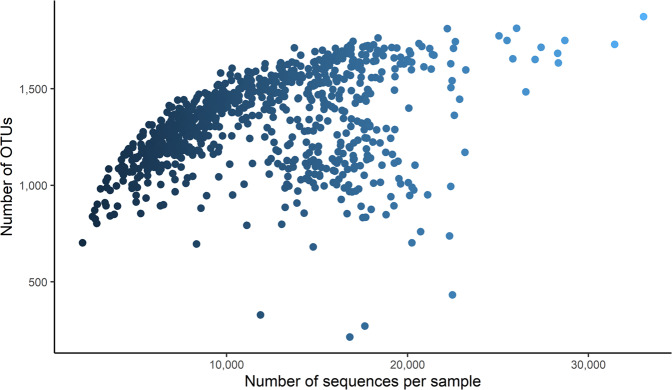
Number of OTUs in terms of the number of sequences per sample.

The abundance table, delivered by FROGS, had 795 samples (as rows) and 2,059 affiliated OTUs (as columns), represented by 750 to 168,617 sequences (median of 1,755 DNA sequences per OTU). Rumen samples contained between 214 and 1,874 OTUs (mean of 1,322 ± 239) and between 2,034 and 33,043 sequences per sample (mean of 11,970 ± 5,196) (Fig. 2).

**Fig. 3:**
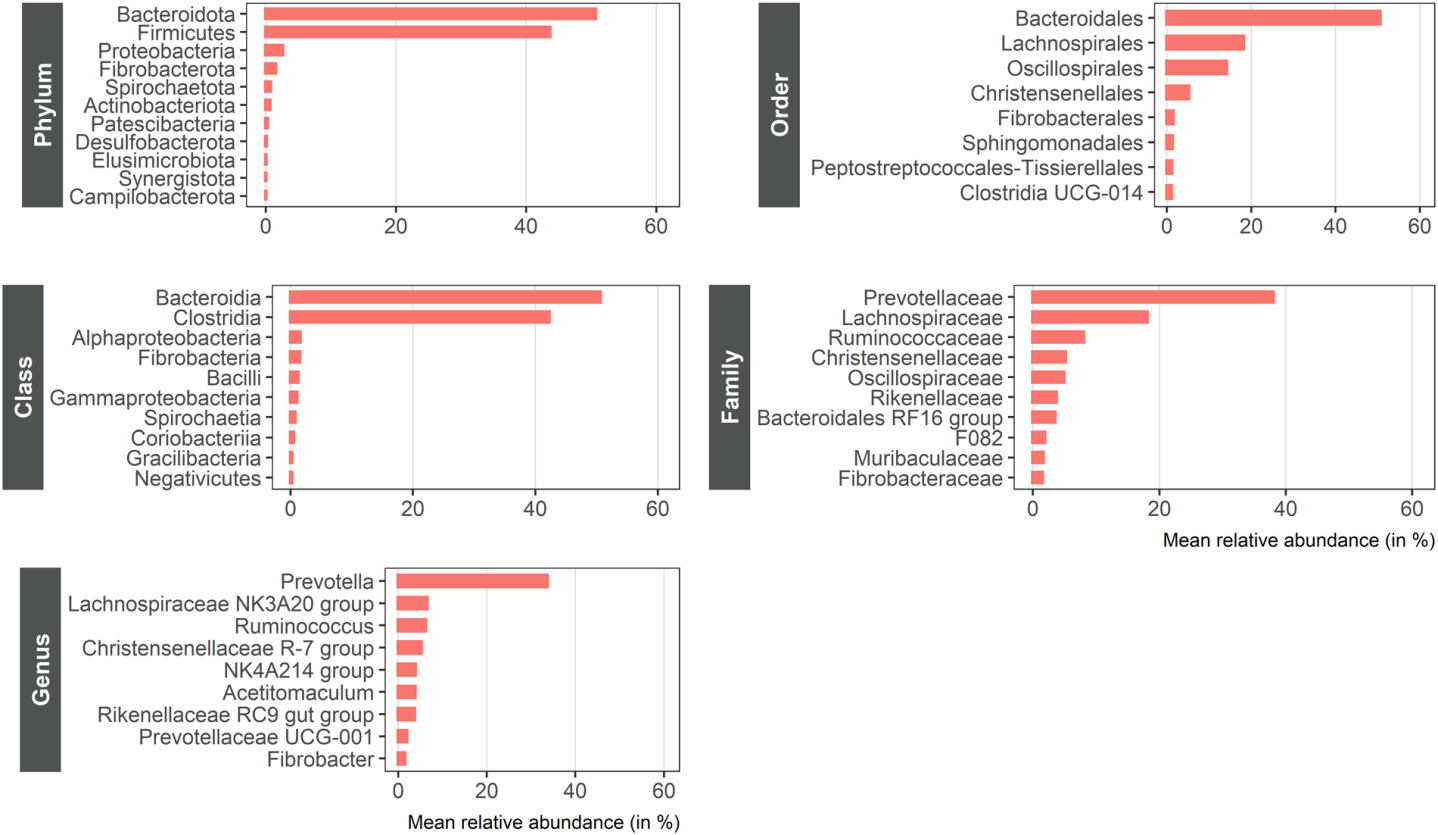
Distribution of taxonomic levels (from phylum to genus) of the 795 ruminal bacterial samples. Top ten most abundant taxa by taxonomic level.

In general, the 2,059 OTUs were assigned to six taxonomic levels with different frequencies of unknown taxonomic affiliation according to OTUs (Table 1). Figure 3 represents the ten most abundant taxa by taxonomic level.

**Table 1:** Frequency and number of operational taxonomic units (OTUs) with unknown taxonomic affiliation.

Taxonomic level	Number of taxons	Number of OTUs with unknown affiliation	Percentage of OTUs with unknown affiliation
Phylum	11	0	0
Class	17	0	0
Order	39	20	1.00
Family	56	63	3.00
Genus	112	416	20.2
Species	70	1,936	94.0

The most represented phyla (expressed as % of total sequences) were *Bacteroidota* (50.6%), *Firmicutes* (43.6%), *Proteobacteria* (2.58%) and *Fibrobacterota* (1.47%). Additionally, the most abundant families were *Prevotellaceae* (37.9%), *Lachnospiraceae* (18.1%), *Ruminococcaceae* (8.97%), *Christensenellaceae* (5.13%), *Oscillospiraceae* (4.86%), and *Rikenellaceae* (3.66%), and the most abundant genera were *Prevotella* (33.6%), *Lachnospiraceae_NK3A20_group* (6.49%), *Ruminococcus* (6.10%), and *Christensenellaceae_R-7_group* (5.12%).

### Milk sampling and traits

Milk samples are official daily records obtained under the genetic selection program of the Lacaune breed in France. The procedure was performed at the Experimental Unit of La Fage (INRAE, France). The ewes were sampled only once during morning and afternoon milking. From these milk samples we quantified daily milk yield (MY). In addition, samples were analyzed at the Interprofessional Milk Analysis Laboratory (Agrolabs, Aurillac, France) to quantify the somatic cell count using a Fossomatic cell counter (Foss, Nanterre, France), and to produce the mid-infrared (MIR) spectra of milk using a Milko-ScanTM FT6000 instrument (Foss, Nanterre, France). This method allows to obtain simultaneously several components of interest for milk production. The somatic cell count was transformed to somatic cell score with the following formula: [SCS = 3 + log2(SCC/100,000)], and fat content (FC) and protein content (PC) were obtained from MIR spectra and expressed in grams per 100 ml of milk. Morning and afternoon milking data were weighted on average for daily composition. Moreover, we predicted by pre-established equations the fine profile of milk proteins (Ferrand *et al*.^13^) and fatty acids (Ferrand-Calmels *et al*.^14^) from MIR spectra. These fine milk components were averaged by milk quantity to recover the weighted average of the daily composition. The proteins included in the dataset were four caseins, namely, alpha-S1-casein, alpha-S2-casein, beta-casein and kappa-casein, and two soluble proteins, namely, alpha-lactalbumin and beta-lactoglobulin. The fatty acids included in the dataset were butyric acid (C4:0), caproic acid (C6:0), caprylic acid (C8:0), capric acid (C10:0), lauric acid (C12:0), palmitic acid (C16:0), oleic acid (cis-9 C18:1), rumenic acid (cis-9 trans-11 C18:2) and alpha-linoleic acid (C18:3ω-3).

## Data Records

The sequence read archive (SRA) is publicly available on the National Library of Medicine repository under the BioProject (https://www.ncbi.nlm.nih.gov/bioproject/) called Rumen microbiota of Lacaune dairy sheep from INRAE La Fage (accession number: PRJNA723543)^15^. In addition, the raw data is available in Data INRAE repository (10.15454/IOET0P)^16^ under the name “DataINRAE_795Lacauneewes.tab”. This dataset includes environmental effects and milk yield and fine milk composition traits for each of the 795 ewes. In the dataset the first two columns correspond to the accession number and sample ID to link with the corresponding SRA in the BioProject^15^. In addition, from column 3 to 11 there are environmental effects such as: genetic line (five levels: SCS+ and SCS-, and PERS+ and PERS-, and SOCS2), year of sampling (five levels: 2015, 2016, 2017, 2018, 2019), run of sequencing or batch effect (seven levels) which refers to the laboratory process of sequences, number of lactations (three levels: 2, 3, or 4 lactations), days in milk (DIM: 28 to 133), litter size (two levels: 1 lamb and 2 lambs or more), total number of sequences per sample (seven levels: ≤5,000; >5,000 and ≤10,000; >10,000 and ≤15,000; >15,000 and ≤20,000; >20,000 and ≤25,000; >25,000 and ≤30,000 and >30,000), order of sampling (eight levels) and hour of sampling (two levels: morning or afternoon). From columns 12 to 30, there are 19 phenotypes, such as milk yield (MY), somatic cell score (SCS), fat content (FC), protein content (PC), milk proteins: kappa casein, beta-lactoglobulin, beta casein, alpha-S1 casein, alpha-S2 casein, alpha-lactalbumin, and milk fatty acids: C4:0, C6:0, C8:0, C10:0, C12:0, C16:0, cis-9 C18:1, cis-9 trans-11 C18:2, and C18:3ω-3. A description of milk traits expressed on a daily basis is shown in Table 2.

**Table 2:** Description of daily milk production (ml/animal/day) and composition traits in the dataset.

Trait	Units	Mean	Standard deviation
Milk yield	ml per day	1,946	589
Fat content	g per 100 ml	7.38	1.14
Butyric acid (C4:0)	g per 100 ml	0.25	0.04
Caproic acid (C6:0)	g per 100 ml	0.21	0.03
Caprylic acid (C8:0)	g per 100 ml	0.20	0.03
Capric acid (C10:0)	g per 100 ml	0.73	0.12
Lauric acid (C12:0)	g per 100 ml	0.49	0.09
Palmitic acid (C16:0)	g per 100 ml	1.96	0.37
Oleic acid (cis-9 C18:1)	g per 100 ml	0.81	0.31
Rumenic acid (*cis-9 trans-11* C18:2)	g per 100 ml	0.04	0.02
Alpha-linolenic acid (C18:3ω-3)	g per 100 ml	0.04	0.01
Protein content	g per 100 ml	5.72	0.52
Alpha-S1-casein	g per 100 ml	1.38	0.15
Alpha-S2-casein	g per 100 ml	0.66	0.26
Beta-casein	g per 100 ml	2.10	0.23
Kappa-casein	g per 100 ml	0.45	0.05
Alpha-lactalbumin	g per 100 ml	0.14	0.01
Beta-lactoglobulin	g per 100 ml	0.46	0.05
Somatic cell score (SCS)*		3.11(671)^†^	1.91(2881)^†^

^†^SCC = somatic cell count (values of mean and SD in brackets, expressed as number of cells per ml of milk.

^*^SCS = 3 + log2(SCC/100,000).

## Technical Validation

Rumen sampling was performed from each ewe using a vacuum pump and a medical gastric tube, which allow a qualitative representation of the rumen microbial community in a large number of animals^17,18^. Before amplification, the quality of the extraction was checked by electrophoresis by our laboratory. Then, the amount of DNA was checked after each PCR with a UV spectrophotometer (NanoDrop 8000, Thermo-Fisher, USA). The Genomic and Transcriptomic Platform (INRAE, Toulouse, France) requires a minimum of 30 ng/µl of DNA to start its analysis, which starts at the second PCR. In addition, before sequencing, quality control of the DNA fragments (Fragment Analyzer) of 12 samples per run was performed to verify the size of inserts (approximately 500 bp) and the absence of residual primers and dimer adapters. Between the first and second PCR, there was a difference of approximately 60 bp due to barcoding. The quality management within the platform can be consulted on its website: https://get.genotoul.fr/la-plateforme/get-plage/. The milk samples are official daily records obtained under the genetic selection program of the Lacaune breed in France. The procedure was performed at the Experimental Unit of La Fage (INRAE, France). The milk composition was recovered from the MIR spectra, and the quality of prediction of the equations used to predict protein and fatty acid concentrations are available in the literature^13,14^.

## Data Availability

No custom code was used to generate or process the data described in the manuscript.

## References

[1] Stewart, C.S., Flint, H.J. & Bryant, M.P. in *The Rumen Microbial Ecosystem* (ed. Hobson, P.N. & Stewart, C.S.) Ch 2. (Springer Netherlands, 1997).

[2] Denman SE, Morgavi DP, McSweeney CS (2018). Review: The application of omics to rumen microbiota function. Animal.

[3] Quast C (2012). The SILVA ribosomal RNA gene database project: improved data processing and web-based tools. Nucleic Acids Res..

[4] Martinez Boggio G, Meynadier A, Daunis-i-Estadella P, Marie-Etancelin C (2021). Compositional analysis of ruminal bacteria from ewes selected for somatic cell score and milk persistency. PLoS ONE.

[5] Rupp R, Lagriffoul G, Astruc JM, Barillet F (2003). Genetic parameters for milk somatic cell scores and relationships with production traits in French Lacaune dairy sheep. J. Dairy Sci..

[6] Rupp R (2009). Response to somatic cell count-based selection for mastitis resistance in a divergent selection experiment in sheep. J. Dairy Sci..

[7] Rupp R (2015). A point mutation in suppressor of cytokine signalling 2 (Socs2) increases the susceptibility to inflammation of the mammary gland while associated with higher body weight and size and higher milk production in a sheep model. PLoS Genet..

[8] Liu Z, Lozupone C, Hamady M, Bushman FD, Knight R (2007). Short pyrosequencing reads suffice for accurate microbial community analysis. Nucleic Acids Res..

[9] Andersson AF (2008). Comparative analysis of human gut microbiota by barcoded pyrosequencing. PLoS ONE.

[10] Escudié F (2018). FROGS: Find, Rapidly, OTUs with Galaxy Solution. Bioinformatics.

[11] Mahé F, Rognes T, Quince C, de Vargas C, Dunthorn M (2015). Swarm v2: highly-scalable and high-resolution amplicon clustering. PeerJ.

[12] Bokulich NA (2013). Quality-filtering vastly improves diversity estimates from Illumina amplicon sequencing. Nat. Methods.

[13] Ferrand, M. *et al*. Determination of protein composition in milk by mid-infrared spectrometry. In: *Proceedings of the 38th ICAR annual meeting* (2012).

[14] Ferrand-Calmels M (2014). Prediction of fatty acid profiles in cow, ewe, and goat milk by mid-infrared spectrometry. J. Dairy Sci..

[15] (2021). NCBI Sequence Read Archive.

[16] Martinez Boggio G (2021). Portail Data INRAE.

[17] Henderson G (2013). Effect of DNA extraction methods and sampling techniques on the apparent structure of cow and sheep rumen microbial communities. PLoS ONE.

[18] Ramos-Morales E (2014). Use of stomach tubing as an alternative to rumen cannulation to study ruminal fermentation and microbiota in sheep and goats. Anim. Feed Sci. Technol..

